# Credibility-based knowledge graph embedding for identifying social brand advocates

**DOI:** 10.3389/fdata.2024.1469819

**Published:** 2024-11-20

**Authors:** Bilal Abu-Salih, Salihah Alotaibi, Manaf Al-Okaily, Mohammed Aljaafari, Muder Almiani

**Affiliations:** ^1^King Abdullah II School of Information Technology, The University of Jordan, Amman, Jordan; ^2^Information Systems Department, College of Computer and Information Sciences, Imam Mohammad Ibn Saud Islamic University (IMSIU), Riyadh, Saudi Arabia; ^3^School of Business, Jadara University, Irbid, Jordan; ^4^School of Business, The University of Jordan, Amman, Jordan; ^5^Management Information System, School of Business, King Faisal University, Al-Ahsa, Saudi Arabia; ^6^Management Information Systems Department, Gulf University for Science & Technology, Kuwait City, Kuwait

**Keywords:** online Customer Engagement, brand advocates detection, Knowledge Graphs, knowledge graph embedding, social credibility, social media analysis

## Abstract

Brand advocates, characterized by their enthusiasm for promoting a brand without incentives, play a crucial role in driving positive word-of-mouth (WOM) and influencing potential customers. However, there is a notable lack of intelligent systems capable of accurately identifying online advocates based on their social interactions with brands. Knowledge Graphs (KGs) offer structured and factual representations of human knowledge, providing a potential solution to gain holistic insights into customer preferences and interactions with a brand. This study presents a novel framework that leverages KG construction and embedding techniques to identify brand advocates accurately. By harnessing the power of KGs, our framework enhances the accuracy and efficiency of identifying and understanding brand advocates, providing valuable insights into customer advocacy dynamics in the online realm. Moreover, we address the critical aspect of social credibility, which significantly influences the impact of advocacy efforts. Incorporating social credibility analysis into our framework allows businesses to identify and mitigate spammers, preserving authenticity and customer trust. To achieve this, we incorporate and extend DSpamOnto, a specialized ontology designed to identify social spam, with a focus on the social commerce domain. Additionally, we employ cutting-edge embedding techniques to map the KG into a low-dimensional vector space, enabling effective link prediction, clustering, and visualization. Through a rigorous evaluation process, we demonstrate the effectiveness and performance of our proposed framework, highlighting its potential to empower businesses in cultivating brand advocates and driving meaningful customer engagement strategies.

## 1 Introduction

The rise of Web 2.0 has transformed online users from mere consumers to active content producers, leading to a notable variation in content quality. Online Social Networks (OSNs) have emerged as a powerful tool for information diffusion across various domains. Consequently, understanding and comprehending the content shared on OSNs have become crucial areas of research. Identifying reputable social content is a time-consuming process but holds significant importance in fields like politics (Ogunmokun and Timur, 2022), e-commerce (Shen, 2023), e-learning (Ajibade and Zaidi, 2023), healthcare (Afful-Dadzie et al., 2023), travel (Liu et al., 2023), etc. As a result, online Customer Engagement (CE) has emerged as a critical element of business success strategies (Han and Anderson, 2022). Online CE goes beyond understanding customer buying behavior and aims to comprehend customers' attitudes toward a brand or company. By providing tailored experiences, businesses seek to turn customers into advocates through OSNs (Oh et al., 2017; Van Doorn et al., 2010). Advocates share positive experiences, influencing others to become customers and potentially becoming brand loyalists themselves (Kartajaya et al., 2016; Kim and Hwang, 2022). This process creates a cycle of advocacy and evangelism, where satisfied customers continuously promote the brand to others. Businesses recognize the value of engaged customers and seek ways to capture positive feedback to convert happy consumers into brand advocates and attract new customers (Lee and Kim, 2022; Nasr et al., 2017; Song and Kim, 2022; Mansoor and Paul, 2022). Consequently, fostering online advocacy and cultivating evangelists have become key objectives for businesses operating in the dynamic landscape of online CE.

Online customer advocacy has emerged as a strategic approach to adopt favorable and reciprocal relationships between companies and their consumers, leading to improved organization (Dutta et al., 2021; Malesev and Cherry, 2021). This concept revolves around encouraging customers to enthusiastically recommend products or services to others, resulting in positive word-of-mouth (WOM). Despite its significance, the motivations behind consumer advocacy behaviors have not been extensively studied, and there is a lack of intelligent systems capable of identifying online advocates based on their social interactions with brands. This constraint highlights the necessity for novel strategies to overcome this obstacle. Knowledge Graphs (KGs) have garnered considerable attention in both the industry and academic sectors due to their ability to present structured and factual representations of human knowledge. This enables them to effectively tackle intricate real-world challenges across diverse domains (Abu-Salih, 2021). KGs possess the potential to significantly augment CE by offering a holistic comprehension of customers, their preferences, and their interactions with a brand.

Furthermore, social credibility plays a crucial role in social customer advocacy as it directly influences the effectiveness and impact of advocacy efforts. Customers are more likely to trust and consider recommendations from individuals they perceive as credible. When brand advocates have a high level of social credibility, their advocacy messages carry more weight and are seen as reliable, increasing the likelihood that others will follow their recommendations. However, the existing body of research on social customer advocacy often overlooks the critical aspect of credibility, which plays a pivotal role in shaping consumer perceptions and behaviors. Social credibility encompasses the trustworthiness, expertise, and reliability of information and sources in the online environment (Abu-Salih et al., 2022). In fact, the integration of credibility into the study of social customer advocacy is relatively scarce. By incorporating social credibility analysis into their advocacy strategies, brands can effectively identify and mitigate the presence of spammers, thereby preserving their authenticity, reputation, and the trust of their customers. This ultimately contributes to the success and effectiveness of their advocacy initiatives in the long run.

In response to the above indicated issues, this study aims to contribute to the growing body of literature by proposing a framework that leverages KG construction and embedding techniques to identify brand advocates. By harnessing the power of KG, the proposed framework seeks to enhance the accuracy and efficiency of identifying and understanding brand advocates, thereby offering valuable insights into the dynamics of customer advocacy in the online realm. To address the social credibility problem, we incorporate and extend DSpamOnto (Al-Hassan et al., 2023), a specialized ontology meticulously crafted for the purpose of identifying social spam in microblogging, with a particular emphasis on targeting a specific domain. The ontology's primary objective is to enhance the comprehension of social spammers' behavior within a focused domain context, encompassing areas like social commerce. While traditional graph representation methods, such as adjacency matrices, can be used to solve graph-related problems, mapping the entire graph or its nodes into a vector space has gained significant attention in the scientific community (Fanourakis et al., 2023). Therefore, we have employed various cutting-edge embedding techniques to map the KG into a low-dimensional vector space. To gauge the effectiveness and performance of the incorporated embedding models, we have designed a comprehensive evaluation protocol and utilized key evaluation metrics to quantify their capabilities. These resultant KG embeddings serve as the foundation for conducting several essential tasks, including link prediction, clustering, and visualization.

This paper differentiates itself from other seminal works as follows; (i) Unlike many existing studies in the field, our research uniquely combines the power of KGs, KGEs, and social credibility analysis to identify and understand brand advocates. This multi-faceted approach allows us to capture a more holistic view of customer advocacy, which is often overlooked in other works; (ii) Our study leverages transductive learning, which is not commonly used in similar works. This approach allows us to make predictions about specific instances that are present during training but lack labels, enhancing the model's ability to generalize from known instances to unknown ones; and (iii) We incorporate and extend DSpamOnto, a specialized ontology for identifying social spam in microblogging. This is a novel approach that enhances our understanding of spammers' behavior within a focused domain context, which is not typically addressed in similar works.

This paper is structured as follows: In Section 2, an extensive review of relevant literature pertaining to the context of this research is presented. Section 3 offers a comprehensive discussion of the overall methodology, elucidating the proposed framework and the constituent modules in detail. The experimental procedures undertaken in this study are expounded upon in Section 4, along with a comprehensive explanation of the evaluation mechanism and the implemented tasks. Section 5 discusses the key contributions of this paper and its existing limitations. Finally, Section 6 encapsulates the conclusions drawn from this research endeavor and delineates potential avenues for future research in this domain.

## 2 Theoretical background

### 2.1 Social customer advocacy

CE and brand advocacy have become critical focal points in the marketing landscape, driven by the widespread adoption of online social networks (Aljarah et al., 2022). The rise of social media platforms has transformed the way businesses and consumers interact, providing unprecedented opportunities for dynamic conversations and interactions (Abu-Salih et al., 2021b). Positive customer word-of-mouth (WOM) has emerged as a significant component of this discourse, encompassing actions by satisfied customers who actively promote and recommend products or services to their social circles (Ginting et al., 2023). Positive WOM wields substantial influence as it emanates from trusted sources and significantly impacts consumer decision-making processes. Conversely, negative WOM can have adverse effects on a company's reputation and sales performance. Therefore, understanding and effectively managing the spectrum of customer WOM, including both positive and negative expressions, is of paramount importance for businesses seeking to cultivate a positive brand image and engage with their target audience (Chen et al., 2023; Hancock et al., 2023).

Brand advocacy plays a key role in CE by proactively promoting and supporting customers' interests and requirements within an organizational context (Wong, 2023). Advocates champion the voices of customers, ensuring their perspectives are considered in decision-making processes and their needs are diligently addressed to enhance the overall customer experience. Effective customer advocacy can be driven by dedicated teams or individual employees who are genuinely passionate about enhancing the customer journey (Campbell et al., 2023). The ultimate goal is to establish a symbiotic relationship between the organization and its customers, characterized by positivity and mutual benefit. Engaging with customers through social media platforms and other online channels fosters a sense of community and cultivates brand loyalty, transforming customers into advocates who actively promote the business to others, thereby amplifying its reach and impact (Aguirre et al., 2023; Bazi et al., 2023).

### 2.2 Social credibility

Online social platforms have become instrumental in circulating information, supporting movements of dissent and protest, and exposing corrupt practices (Bhatia et al., 2023). However, along with these advantages, the absence of strict gatekeeping mechanisms has also opened the door to fraudulent activities, defamation, and the rapid spread of rumors, which can significantly damage the reputation of both organizations and individuals (Abu-Salih et al., 2022, 2021b; Wongthontham and Abu-Salih, 2018). The prevalence of false information and malicious activities on these platforms poses significant challenges, as they degrade the overall quality of experience for members of these virtual communities. It is crucial to address these challenges and find effective strategies to ensure the integrity and trustworthiness of online social networks while preserving the principles of freedom of expression and open communication.

Social credibility plays a crucial role in social customer advocacy as it directly influences the effectiveness and impact of advocacy efforts. Customers are more likely to trust and consider recommendations from individuals they perceive as credible (Wang et al., 2022). When brand advocates have a high level of social credibility, their advocacy messages carry more weight and are seen as reliable, increasing the likelihood that others will follow their recommendations. Their recommendations and positive experiences can persuade potential customers to try a brand's offerings and develop a positive attitude toward the brand (Lavoye et al., 2023). Social credibility enhances the reach and impact of advocacy efforts. Advocates with high credibility tend to have larger social networks and a greater level of influence within those networks. Their advocacy messages are more likely to be shared, liked, and followed by others, leading to a wider dissemination of positive brand information.

At the same time, the analysis of social credibility is not only important for identifying credible advocates but also for detecting spammers or individuals who engage in deceptive practices. For brands, identifying and mitigating the presence of spammers is crucial for maintaining the integrity of their advocacy efforts and protecting their reputation. Spammers often engage in activities that are deceptive or manipulative, such as posting fake reviews or engaging in unethical promotional tactics. By detecting and filtering out spammers, brands can ensure that their advocacy efforts are genuine and authentic, maintaining the trust and credibility they have built with their audience (Jashmine et al., 2022). Furthermore, spammers can dilute the impact of genuine advocacy efforts by creating noise and clutter in online discussions. By identifying and removing spammers, brands can ensure that their advocacy messages are more visible and impactful, reaching their intended audience without interference.

### 2.3 Domain knowledge graphs

Domain-specific KGs are specific KGs that concentrate on acquiring and organizing data within a particular topic or industry. These KGs act as potent stores of semantic relationships and structured data, facilitating sophisticated knowledge discovery, reasoning, and data analysis within the intended topic. They are described as “explicit conceptualization to a specific subject-matter domain represented in terms of semantically interrelated entities and relations” (Abu-Salih, 2021). In addition to their application in various industries and domains, domain-specific KGs have emerged as a powerful tool in research and academia. Scholars and researchers are increasingly recognizing the significance of domain-specific KGs in organizing, managing, and exploring large volumes of specialized knowledge within specific fields (Ren et al., 2023; Zhong et al., 2023; Zhu et al., 2023). These knowledge graphs serve as a valuable resource for experts, students, and enthusiasts, offering a comprehensive and interconnected view of the domain's key concepts, historical developments, and ongoing research. Further, domain-specific KGs foster collaboration and knowledge sharing among researchers, enabling them to build upon each other's work and contribute to the collective understanding of their respective domains. As research continues to advance, domain-specific knowledge graphs are expected to play an instrumental role in accelerating breakthroughs, fostering innovation, and shaping the future of specialized knowledge acquisition and dissemination (Zhou et al., 2022).

### 2.4 Related works

The integration of artificial intelligence (AI) in enhancing CE through social media interactions has proven to be a transformative approach for businesses. Leveraging the vast amount of social data available, AI algorithms can analyze customer interactions, sentiments, and preferences at scale, enabling brands to gain deep insights into consumer behavior and preferences (Gao et al., 2023). AI-powered systems can quickly identify patterns, trends, and emerging issues from customer feedback, allowing businesses to respond promptly and proactively to customer concerns and inquiries. Moreover, by understanding the nuances of individual customer interactions, AI can personalize responses and recommendations, fostering a more meaningful and personalized customer experience (Menidjel et al., 2022). The integration of AI in customer-brand interactions not only streamlines customer support processes but also enhances customer loyalty and satisfaction (Bedi et al., 2022). As AI technology continues to advance, its potential impact on CE is likely to grow, shaping the future landscape of customer-brand interactions and driving innovation in the realm of social media marketing (Haleem et al., 2022).

In the context of online engagement, domain-specific KGs play an essential role in enhancing our understanding of customer behavior, preferences, and sentiments within a specific industry or market (Pai et al., 2022). However, the existing research on customer advocacy has largely focused on examining specific social media metrics, such as the number of likes, shares, or comments on-brand content, to gauge CE. While these metrics provide valuable insights, they often lack the depth and context required to truly understand the nature of the conversations between brands and their customers. As a result, there has been a notable gap in the literature concerning a more comprehensive analysis of the broader social conversations and interactions that occur between brands and their customers. Our study addresses this gap by proposing a novel approach that goes beyond superficial metrics and explores the textual content of the conversations between brands and customers.

## 3 Methodology

**Figure 1:**
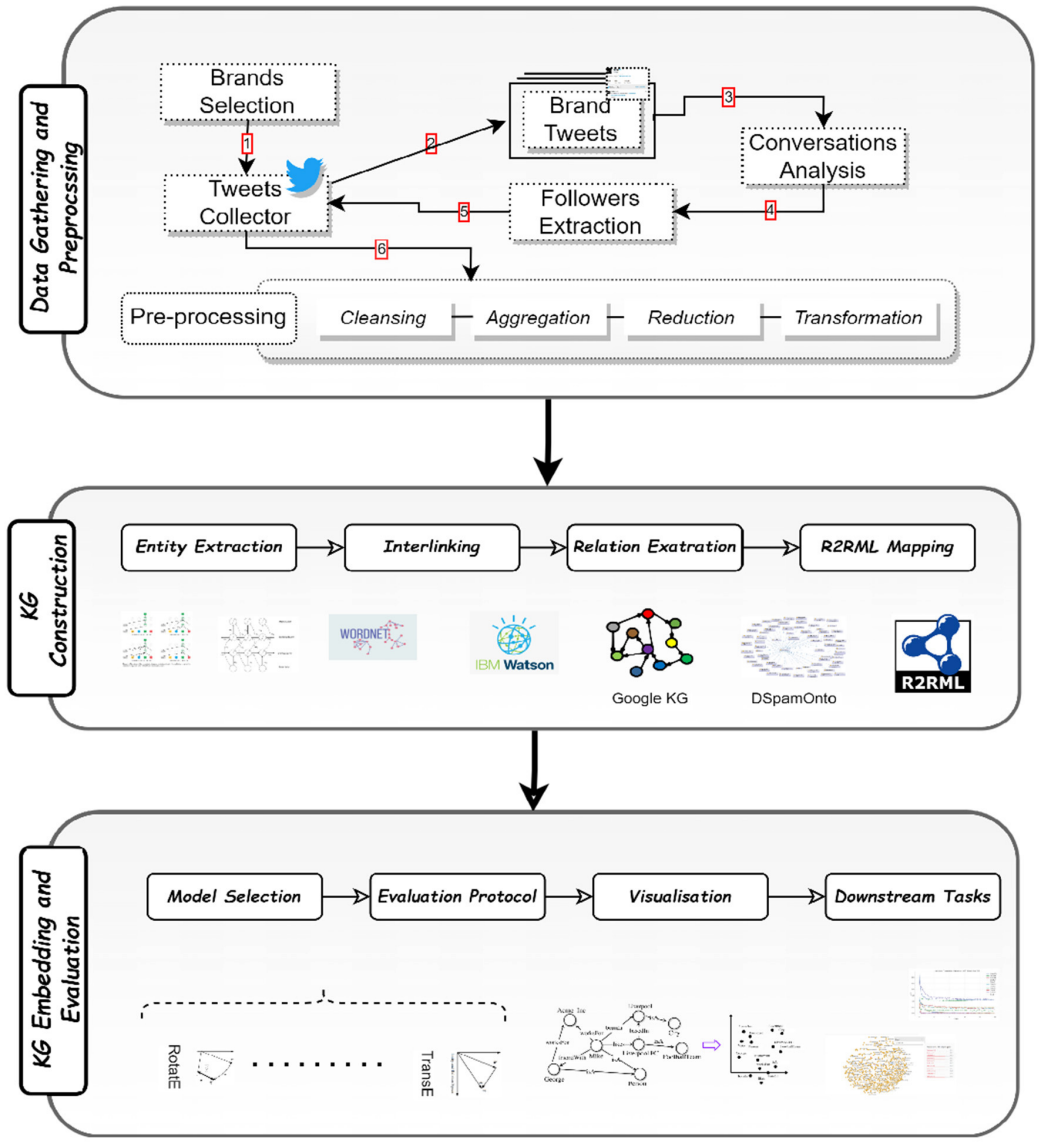
A diagram of the proposed framework.

The primary modules included in this study are covered in this section. Following a summary of the dataset that was gathered, the methods utilized for KG building and KG embedding are presented. A schematic depiction of the suggested framework is shown in Figure 1.

### 3.1 Data collection and preparation

Twitter has emerged as a crucial platform for customer advocacy, boasting unique characteristics that have contributed to its widespread adoption as a powerful social media platform. Its real-time and instantaneous communication capabilities allow customers to engage in immediate exchanges, sharing their experiences, opinions, and recommendations about various brands and products. This real-time nature renders Twitter a dynamic space where customers can passionately advocate for a brand or express their dissatisfaction, thereby exerting a considerable influence on public perception and shaping brand reputation (Boulianne and Larsson, 2023; Charoenthansakul and Natee, 2023; Lievonen et al., 2023).

Figure 1 depicts the comprehensive process employed to extract social data and metadata pertaining to brands and customers for our study. Specifically, we carefully select nice official Australian brand Twitter accounts as the primary focus of our analysis. To gather relevant data, Twitter APIs are utilized to collect the tweets posted by these brand accounts. Subsequently, we conduct an in-depth analysis of these tweets to extract the conversations that transpire between the brands and their customers. To ensure the reliability and quality of the data, we apply rigorous filtering techniques to eliminate any poor or limited conversations. This step is crucial in guaranteeing that our proposed model operates on customers with substantial and meaningful brand dialogues. By focusing on conversations with sufficient depth and relevance, we can derive more accurate insights into customer advocacy and its impact on brand perception. Having identified the conversations, we proceeded to acquire the Twitter accounts of the participating customers. Through this process, we obtain both their social data, represented by the tweets they post, and their metadata, encompassing relevant account information. This comprehensive dataset allows us to gain a comprehensive understanding of the interactions and engagements between the brand and its customers on social media. To further enhance the accuracy of our analysis, we conduct manual evaluations of the contents of these customer tweets. This meticulous manual analysis enables us to identify advocate customers, who exhibit positive opinions and sentiments toward the brand. These advocate customers play a crucial role in amplifying the brand's reputation through positive WOM practices. By recommending the brand's products or services and expressing favorable sentiments in their tweets, these advocates significantly influence the perceptions and preferences of their social circles.

### 3.2 KG construction

KG Construction to model advocate customers involves constructing a KG that captures the relationships and interactions between customers, brands, and products. This enables a deeper understanding of customer advocacy and its implications for brand management and decision-making. Also, the KG contains various other entities that capture the social characteristics of customers, brands and products. The following sections describe the technicalities that are included in constructing the intended KG.

#### 3.2.1 Entity and relation extraction

In the context of customer advocacy, entity extraction plays a crucial role in identifying and capturing key pieces of information from unstructured textual data. This process involves recognizing and extracting relevant entities that are associated with customer advocacy activities, such as brand names, product names, customer mentions, sentiment expressions, and other pertinent information. By automatically extracting these entities from the text, businesses can gain valuable insights into customer sentiments, brand mentions, and overall customer experiences. Relation extraction is the process of identifying and extracting the semantic relationships between entities mentioned in textual data. In the context of customer advocacy, relation extraction can be used to identify the connections and interactions between brands and customers, such as brand-customer interactions, customer sentiments toward specific products, or the influence of brand advocates on other customers. This helps businesses gain deeper insights into the dynamics of customer advocacy and engagement, enabling them to make more informed decisions and improve their customer advocacy strategies.

To construct the KG, we follow our previously validated framework (Abu-Salih and Alotaibi, 2023) in constructing the KG. In particular, entity extraction in customer advocacy is facilitated by a sophisticated architecture that combines XLNet, BiLSTM, and CRF layers. XLNet, a transformer-based language model, provides bidirectional context information and semantic representations of input text (Yang et al., 2019). It addresses limitations found in BERT by incorporating permutation-based training and the Transformer-XL architecture. The XLNet model processes the input sentence, producing token-level embeddings that capture the semantic meaning of each term. The BiLSTM layer is built on top of the XLNet encoding layer to capture contextual dependencies in both forward and backward directions (Yang et al., 2019). It excels in capturing long-term dependencies in sequential data, making it ideal for tasks like entity recognition in customer advocacy. The hidden states from both the forward and reverse layers are combined to create a comprehensive representation of the input sequence, allowing the model to effectively capture dependencies and make accurate predictions based on the sequential nature of the data. The CRF layer models the sequential dependencies among entity labels. It assigns the most probable label sequence for the input tokens, considering the dependencies between neighboring labels and input features. By explicitly capturing label dependencies, the CRF layer ensures coherent and consistent predictions, leading to more accurate entity extraction results.

#### 3.2.2 Ontology interoperability

The importance of ontology interoperability in this research cannot be overstated, as it facilitates the alignment and integration of the constructed KG with pertinent entities extracted from both existing domain-specific and generic ontologies. This harmonization process ensures that the KG utilized in the study is effectively interconnected and seamlessly integrated within the broader knowledge landscape. To achieve ontology interoperability, several methods are employed in this investigation:

(1) Utilizing Google KG: The research incorporates the Google Knowledge Graph (KG), a vast knowledge base that enhances Google's search engine capabilities. The study employs the Google KG Search API to gather entities and classify them into different classes or types. This API offers access to a diverse range of entities across various domains. By adopting ontology interoperability techniques and integrating the Google KG Search API, the research ensures that the developed ontology is enriched with pertinent entities and harmoniously aligned with existing knowledge sources. This integration significantly enhances the semantic comprehension of CE and brand advocacy, facilitating more precise and comprehensive analysis.

(2) The Natural Language Understanding service by IBM Watson^TM^ (NLU) offers accessible APIs that provide access to a diverse array of linked data resources. These resources include popular vocabularies like Upper Mapping and Binding Exchange Layer (UMBEL), Freebase (a community-curated database containing information on people, places, and things), and YAGO (a high-quality knowledge base), among others. Through these APIs, IBM Watson has the capability to extract entities from social media data, including brand names, customer names, product names, and other pertinent entities. This functionality aids in the identification of key players and entities involved in customer advocacy, facilitating the establishment of relationships between them within the Knowledge Graph (KG).

(3) Entity Alignment in the context of social customer advocacy refers to the process of identifying and establishing equivalent links (URIs) that indicate the same entity or resource across different KGs or ontologies. This alignment is achieved using the owl#sameAs relation, which signifies that the URIs of both the subject and object entities refer to the same resource. The objective of entity alignment is to ensure that common entities, such as brand names, product names, and customer mentions, are represented consistently and standardized across various ontologies.

In social customer advocacy, data from different sources, such as social media platforms and customer feedback channels, may be stored and represented using different ontologies or KGs. These different representations can lead to inconsistencies and difficulties in integrating and analyzing the data coherently. Entity alignment addresses this challenge by mapping equivalent entities from one ontology to another using the owl#sameAs relation. By doing so, the study ensures that entities with different URI representations but referring to the same real-world entity are recognized as equivalent and unified under a single representation.

(4) DSpamOnto Ontology: Incorporating social credibility analysis into advocacy strategies is crucial for brands to maintain authenticity, reputation, and customer trust. By identifying and mitigating the presence of spammers, brands can ensure that their advocacy efforts are genuine and trustworthy. In this context, we incorporate DSpamOnto (Al-Hassan et al., 2023), a domain-specific ontology, serves as a valuable tool for detecting and addressing social spam in microblogging platforms, particularly within specific domains like politics or health. This ontology offers a deeper comprehension of the behavior exhibited by social spammers who concentrate their efforts on a particular domain. By leveraging DSpamOnto, it becomes feasible to identify social spammers based on their domain-specific conduct, which encompasses actions such as posting content that is repetitive or lacks relevance, utilizing misleading information or tactics, and specifically targeting certain domains. Incorporating DSpamOnto into brand advocacy strategies enables brands to maintain a credible and spam-free environment, ultimately supporting the success and effectiveness of their advocacy initiatives.

**Figure 2:**
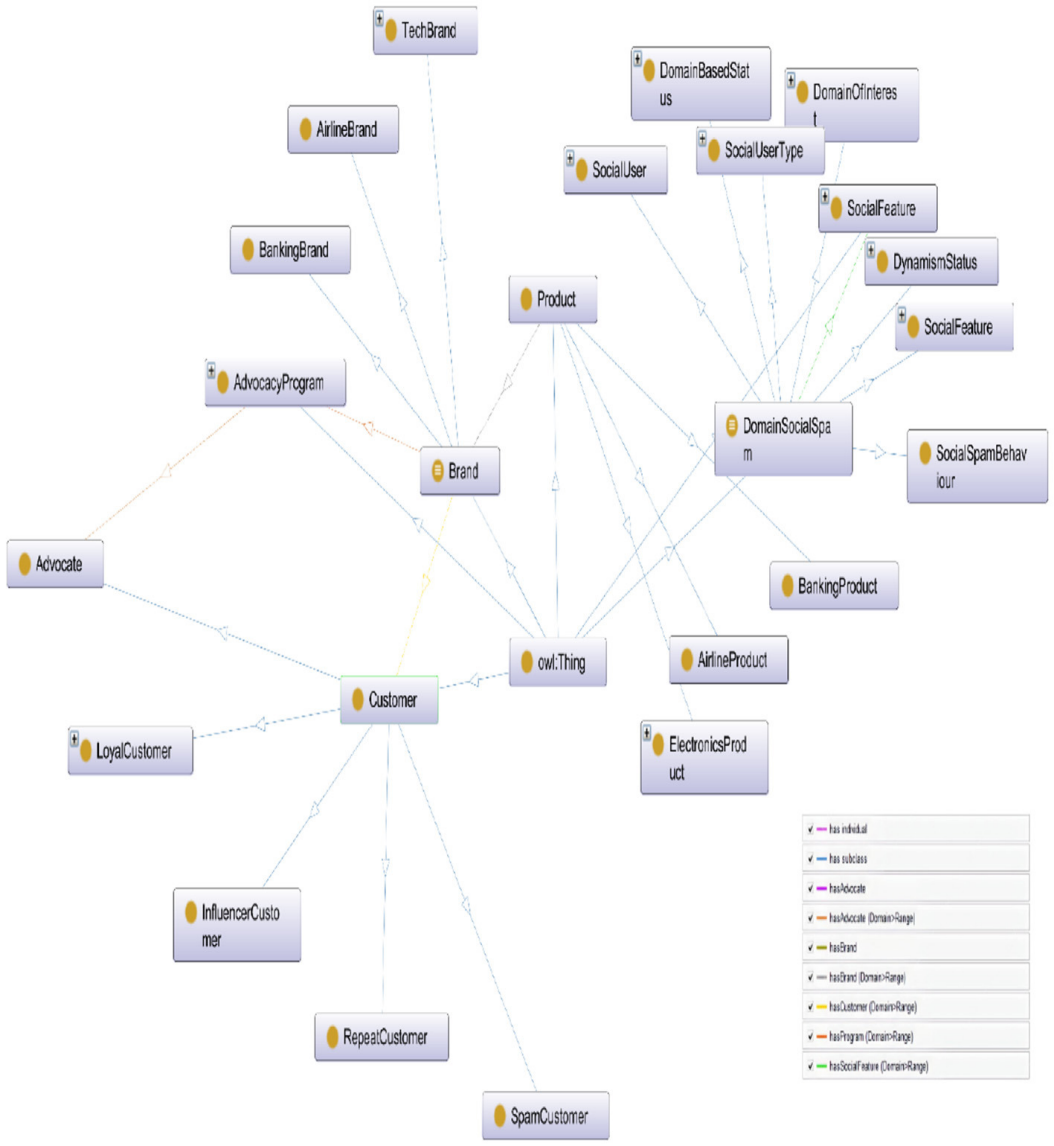
A snapshot of certain classes of the extended DSpamOnto.

We have extended DSpamOnto by adding entities relevant to social credibility, engagement, social influence, and brand advocacy. Ensuring the logical consistency and correctness of an ontology is indeed an important step in ontology design. By utilizing reasoning processes and employing well-known reasoners such as TrOWL, RacerPro, Pellet, HermiT, FaCT++, etc., we can verify the extended DSpamOnto Ontology and ensure that it adheres to the desired specifications. Reasoners play a crucial role in examining the ontology's concepts, properties, instances, and hierarchies, as well as identifying any potential contradictions or inconsistencies. They provide valuable services such as classification and realization, which help in organizing the ontology's concepts into meaningful hierarchies and determining the relationships between them. This process is carried out on DSpamOnto and no contradictory facts were indicated, this implies that the extended ontology is logically consistent and suitable for answering queries related to its semantic concepts, relationships, and instances. Figure 2 demonstrates a snapshot of the extended ontology.

(5) WordNet^1^: We leverage the WordNet lexical database as a valuable resource to enhance the knowledge base and enrich the semantic meaning of terms in the context of social customer advocacy. WordNet is a comprehensive vocabulary lexicon that comprises a collection of interrelated words or terms known as synsets, representing synonyms with similar semantic meanings. By incorporating WordNet into the entity extraction process, we can associate additional semantic-related concepts with a given term, thereby enriching its overall meaning and context.

WordNet offers several benefits when extracting entities relevant to CE and brand advocacy. Firstly, it allows for synonym expansion, enabling us to retrieve synonyms or similar words for specific terms related to customer advocacy. For example, when extracting entities associated with customer satisfaction, WordNet helps us identify synonyms such as satisfaction, pleasure, and contentment. This broadens the scope of captured entities, encompassing a diverse range of concepts linked to customer sentiment. Secondly, WordNet organizes words into hierarchies based on their semantic relationships. This hierarchical structure enables us to extract entities at different levels of abstraction. For instance, exploring WordNet's hierarchy may lead to the identification of broader concepts like marketing, advertising, or brand promotion, which are closely related to the notion of advocacy. By incorporating such hierarchical relationships, we capture a comprehensive set of relevant entities, contributing to a more holistic understanding of customer advocacy. Moreover, WordNet provides valuable information about hyponyms (specific instances or subtypes) and hypernyms (general categories or supertypes) for many words. By analyzing these relationships, we can extract entities that represent specific instances or general categories related to CE and brand advocacy. This allows us to explore both specific and overarching concepts within the domain, providing a more comprehensive perspective on customer advocacy.

By leveraging WordNet during the entity extraction process, our dataset is enriched with a diverse array of relevant entities, synonyms, and semantic relationships. This enhanced dataset facilitates a more comprehensive analysis of CE and brand advocacy, offering deeper insights and a deeper understanding of the domain. As a result, our study benefits from a more nuanced approach to entity extraction, contributing to more accurate and meaningful analyses of social customer advocacy. The incorporation of WordNet's comprehensive vocabulary and semantic relationships empowers our research to uncover valuable patterns, sentiments, and brand perceptions among customers, enhancing the effectiveness of our knowledge graph construction and customer-centric analytics.

**Figure 3:**
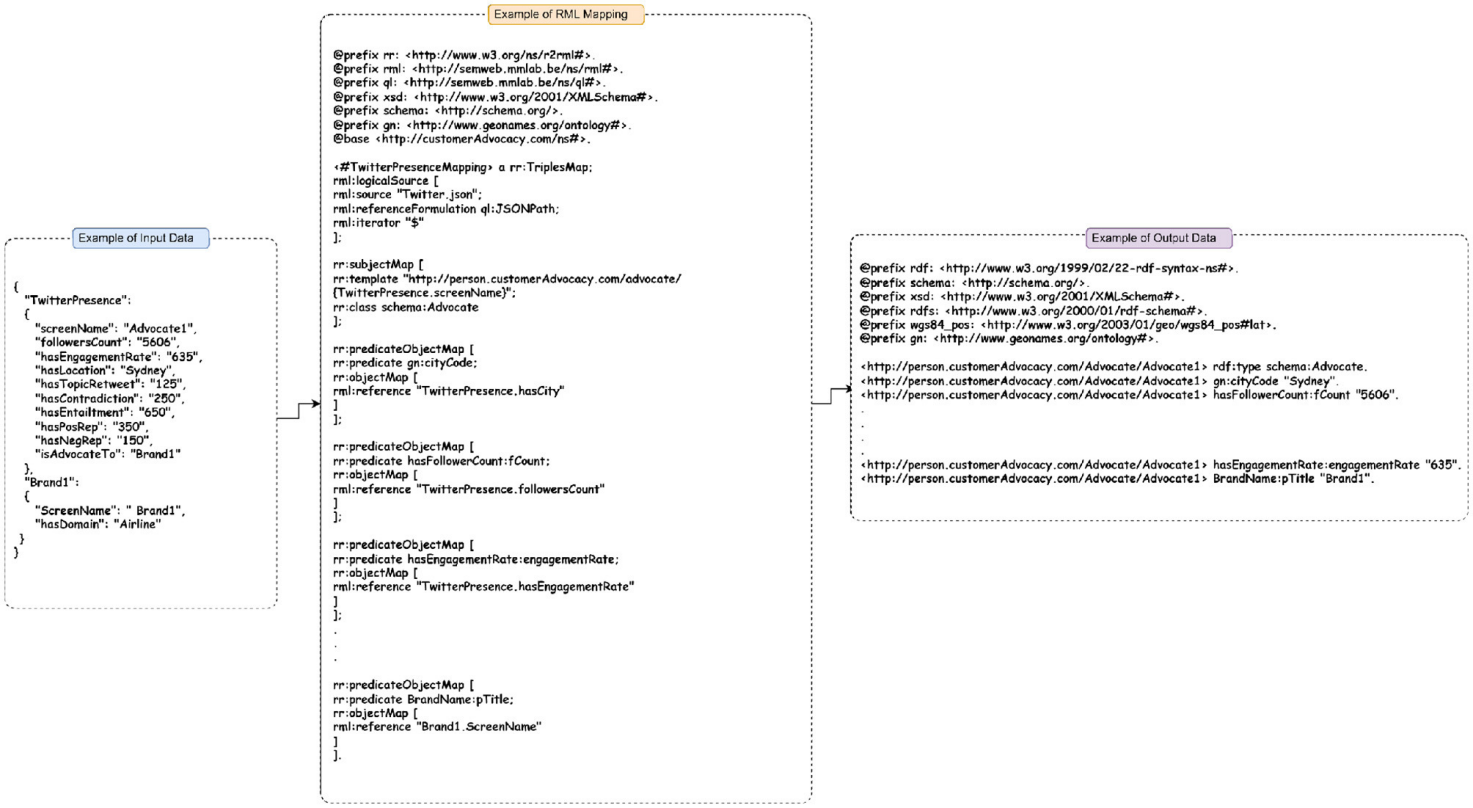
An example of mapping a data source to an RDF incorporating RML technique.

To integrate these data islands, RDF Mapping Language (RML) is used. RML is a language specifically designed for mapping heterogeneous data sources to RDF graphs. RML provides a flexible and expressive framework to define mappings between different data formats and RDF representation (Mishra et al., 2021). With any data source as the input and an RDF graph as the output, RML presents a generic method for mapping various data formats. In RML, there are one or more triple maps that make up the mapping process. Each triple map in RML embodies a logical source (input source), a subject map (describes the process for generating the subject for each logical resource), and a predicate-object-map (describes the predicate and the object map as well as the process for generating the triple's predicate). By leveraging RML, the KG creation process becomes more efficient and scalable. RML enables seamless integration of diverse data sources, handles data transformations and heterogeneity, and aligns the data with relevant ontologies and vocabularies. This facilitates the construction of a comprehensive and semantically rich KG for customer advocacy, allowing for advanced data analysis, querying, and inference over the integrated knowledge. Figure 3 shows an example of mapping an input data source in JSON format to RDF triples for an advocate of Brand1 using RML.

### 3.3 Knowledge graph embedding models

Knowledge Graph Embedding (KGE) is the process of transforming the elements of a KG (entities and relationships) into a lower-dimensional, semantically continuous space. While traditional graph representation methods, such as adjacency matrices, can be used to solve graph-related problems, mapping the entire graph or its nodes into a vector space has gained significant attention in the scientific community (Abu-Salih et al., 2021a). This approach offers scalability and simplifies the resolution of complex real-world graph problems, including KG completion, entity resolution, and link-based clustering, among others. Learning the embedding of a KG involves training a neural architecture on the graph data, typically consisting of three main components: (i) encoding entities as distributed points in the vector space and encoding relationships as vectors or other suitable representations; (ii) utilizing a scoring function or model-specific function to evaluate the effectiveness of the embedding model; (iii) employing an optimization procedure that aims to learn the optimal embedding for the given KG, where the scoring function assigns high scores to positive statements. In the field of KGE, existing literature commonly classifies embedding techniques into two main categories: translation distance models and semantic matching models (So et al., 2022). Translation distance models aim to assess the plausibility of a given fact by measuring the distance between two entities. On the other hand, semantic matching models focus on evaluating the plausibility of facts by considering the latent semantics of entities and relationships within their low-dimensional representations. Within the extensive range of KG embedding models proposed in the literature, this study incorporates a selection of the most popular ones to investigate their effectiveness. the KGE models that are incorporated in this study include: (i) Translating Embedding (TransE) (Zhu et al., 2021); (ii) DistMult (Herlihy and Rudinger, 2021); (iii) Complex Embeddings (ComplEx) (Castillo López et al., 2021); (iv) Holographic Embeddings (HolE) (Goh and Arenas, 2020); (v) Convolutional 2D KG Embeddings (ConvE) (Castillo et al., 2021); (vi) Convolution-based model (ConvKB) (Van Doorn et al., 2010); (vii) RotatE (Sun et al., 2019a); (ix) TransR (Lin et al., 2015); (x) AutoSF (Zhang et al., 2020); and (xi) BoxE (Abboud et al., 2020).

The selection of these embedding models in this study was based on a careful consideration of several factors, including scalability, interpretability, and performance. The incorporated models such as TransE, DistMult, and ComplEx were chosen due to their ability to handle large-scale KGs (Ali et al., 2021; Wang et al., 2018). These models are computationally efficient and can be easily scaled to accommodate larger graphs, making them suitable for large-scale KG embedding tasks. Some models offer more interpretability than others. For instance, TransE interprets relations as translations in the embedding space, providing a clear and intuitive understanding of the relationships between entities (Sun et al., 2019b). Similarly, DistMult and ComplEx provide interpretable embeddings by representing the interaction between entities and relations as a bilinear product or a trilinear Hermitian dot product, respectively (Anelli et al., 2022). The performance of the models on various tasks, such as KG completion and entity resolution, was another crucial factor. Models like RotatE and TransR have shown excellent performance on these tasks in previous studies (Le et al., 2023; Lin et al., 2015), justifying their inclusion in this study. Moreover, the study also aimed to investigate a diverse range of models to gain a comprehensive understanding of KG embedding techniques. This includes models based on different paradigms, such as translation distance models (e.g., TransE, TransR), semantic matching models (e.g., DistMult, ComplEx), and more recent approaches that leverage convolutional operations (e.g., ConvE, ConvKB) or complex-valued vectors (e.g., RotatE).

### 3.4 Knowledge graph embedding model evaluation

In this section, we provide a brief on the performance metrics incorporated in this study to measure the performance of the KG Embedding models as well as on the link prediction task. The following learning-to-rank metrics are commonly used in the literature to measure the performance of KG Embedding models:

- Mean Reciprocal Rank (MRR): is a function that computes the mean of the reciprocal of elements embodied in a vector of rankings. It is used as a measure to evaluate the system performance against the retrieved elements. The formal definition of MRR is:

(1) $$\begin{array}{l} \text{MRR}=\frac{1}{\left|Q\right|}\overset{\left|Q\right|}{\underset{i=1}{\sum }}\frac{1}{\text{rank}{\left(s,p,o\right)}_{i}} \end{array}$$

where *rank*(*s, p, o*)_*i*_ refers to the rank of a positive element *i* against a list of negative elements, *T* is a set of test triples and (*s, p, o*) is a triple ∈*T*. MRR evaluates the model's ability to rank the correct triple (head entity, relation, tail entity) higher than incorrect triples. It measures the accuracy of the model's ranking in capturing the true KG facts.

- Mean Rank (MR): refers to the mean rank of the correct test facts/triples embodied in a vector of rankings (i.e., the average of the predicted ranks). MR can be computer as follows:

(2) $$\begin{array}{l} MR=\frac{1}{\left|T\right|}\overset{\left|Q\right|}{\underset{i=1}{\sum }}\mathrm{rank}{\left(s,p,o\right)}_{i} \end{array}$$

MR provides a direct measure of how well a KG embedding model ranks the correct triple (head entity, relation, tail entity) among all possible alternatives. It represents the average position of the correct triple in the ranked list. A lower MR value indicates better ranking performance.

- Adjusted Mean Rank (AMR): This metric is calculated by summing the reciprocal of the rank multiplied by the reciprocal of the number of correct tail entities for each query. This value is then divided by the total number of queries to obtain the average adjusted mean rank as follows:

(3) $$\begin{array}{l} \text{AMR}=\frac{\sum_{i=1}^{n}\left(\frac{1}{{\text{rank}}_{i}^{*}}\times {\text{num\_correct}}_{i}\right)}{\text{num\_queries}} \end{array}$$

Where *rank*_*i*_ is the rank of the correct tail entity for query i, *num correct*_*i*_ is the number of correct tail entities for query i, *num*_*queries*_ is the total number of queries.

- Hits@N: indicates the number of elements in the ranking vector retrieved from the model is positioned in the top ( *N*) locations. *Hits@N* can be defined as follows:

(4) $$\text{Hits}@\text{N=}\{\begin{array}{cc} \sum_{i=1}^{\left|Q\right|}1, & rank{\left(s,p,o\right)}_{i}\leq N\\ \text{0}, & \text{ otherwise } \end{array}$$

*Hits@N* provides the probability that the correct results appear on the top where the proportion of the ranks does not exceed *N*. When *N* = 1 it checks if the target test fact can be correctly predicted from the first attempt. Hits@N allows evaluation at different levels of precision depending on the chosen value of N. By considering different values of N, such as Hits@1, Hits@3, or Hits@10, it provides a range of precision levels to assess the model's ability to rank the correct triple within the top-N positions.

- Cross-Entropy Loss: is a commonly used loss function for classification problems. Cross-Entropy Loss measures the dissimilarity between the predicted probability distribution and the actual distribution. In the context of our study, it quantifies the difference between our model's predictions and the actual outcomes.

The Cross-Entropy Loss is defined as:


(5) $$L(y,\hat{y})=-\frac{1}{N}\sum_{i=1}^{N}y_{i}\log({\hat{y}}_{i})+(1-y_{i})\log(1-{\hat{y}}_{i})$$

Where: *y* is the actual outcome, ŷ is the predicted outcome, *N* is the total number of observations.

A lower Cross-Entropy Loss indicates that our model's predictions are close to the actual outcomes, implying better model performance. Conversely, a higher Cross-Entropy Loss suggests that the predictions are far from the actual outcomes, indicating poorer model performance.

Another key metric is used to evaluate the performance of the link prediction task. The metric is composed of the three well-known measures, namely: Precision, Recall and F-Score, that are commonly used in classification tasks. Further discussion on these metrics and their usage is depicted in Section 4.4.

The selection of these evaluation metrics has been carried out using a thorough process. Firstly, the chosen metrics needed to be directly relevant to our research objectives. For instance, if our objective was to predict a binary outcome, we opted for metrics such as accuracy, precision, recall, and the F1 score. These metrics are standard in binary classification tasks and provide a comprehensive view of the model's performance. Secondly, we prioritized interpretability. We aimed to select metrics that are easily understandable and meaningful to both researchers and stakeholders. For example, accuracy, which represents the proportion of correct predictions, is straightforward and widely understood. Thirdly, we considered the robustness of the metrics to imbalanced data. In cases where our dataset was imbalanced, we used metrics such as the F1 score. These metrics are less sensitive to imbalanced data compared to accuracy. Lastly, we needed metrics that allowed us to compare different models effectively. For example, the MSE and RMSE are useful for comparing regression models, while the log loss is beneficial for comparing probabilistic classifiers.

## 4 Experimental results

### 4.1 Dataset exploration

The utilization of Twitter as a customer service platform has yielded significant results, with 85% of small and medium businesses (SMB) acknowledging its impact (Sayce, 2022). This underscores the need for continuous implementation of advanced tools to comprehend customers better and derive the desired added value. In this study, the focus lies on online CE facilitated by the Twitter platform, utilizing a comprehensive social dataset. Figure 1 depicts the step-by-step process employed to extract social data and metadata pertaining to brands and customers. Specifically, nine official Australian brand Twitter accounts were selected, and their tweets were collected through Twitter APIs. These tweets were subsequently analyzed to extract meaningful conversations between the brands and customers. Careful filtration was applied to eliminate subpar and limited conversations, ensuring that the proposed model operates on customers engaged in meaningful brand dialogue. The Twitter accounts of these customers, along with their associated social data (tweets) and metadata (account information), were acquired. The contents of these customers' tweets were preliminarily analyzed using IBM Watson NLU to identify customers who expressed positive sentiments and recommendations toward the brand. Those customers and their social data and metadata are then manually analyzed, thereby extracting advocates who actively promote and recommend a brand's products or services. Cohen's kappa agreement metric is used in this experiment for assessing the inter-rater reliability between the two annotators. It takes into account the agreement observed between the experts' labels and adjusts for the agreement that would be expected by chance. Cohen's kappa ranges from −1 to 1, with values closer to 1 indicating higher agreement beyond chance. It is commonly used for binary or categorical labeling tasks. Table 1 shows certain statistics of the collected dataset.

**Table 1:** The number of labeled advocates and spammers for each brand.

**Brand**	**#advocates**	**#spammers**
Brand1	1,171	102
Brand2	291	38
Brand3	981	106
Brand4	2,607	77
Brand5	4,634	913
Brand6	53	16
Brand7	3,179	386
Brand8	398	68
Brand9	1,176	171

Table 1 also shows the number of spammers detected in the dataset. The spammers are identified based on the DSpamOnto ontology (Al-Hassan et al., 2023) that is incorporated to extract domain-specific social spam from the dataset.

### 4.2 XLNet-BiLSTM-CRF implementation

The XLNet-BiLSTM-CRF architecture, as detailed in our previous work (Abu-Salih and Alotaibi, 2023), has been meticulously implemented with careful consideration for optimizing the training process. Firstly, we increased the complexity and depth of the XLNet-Base model by employing a larger variant with 24 layers, 1,024 hidden units, and 16 attention heads. This augmentation allows the model to capture intricate patterns and relationships within the data, leading to improved representation learning. To handle longer sequences and capture more contextual information, we adjusted the max-seq-length parameter to 256. By doing so, the model can effectively process and encode more comprehensive textual contexts, resulting in enhanced understanding and inference capabilities. To strike a balance between computational efficiency and model performance, we increased the train-batch-size, eval-batch-size, and predict-batch-size to 64, enabling larger mini-batches for training and evaluation. This adjustment facilitates stable and accurate gradient estimation, contributing to improved convergence and generalization. For optimizing the learning process, we fine-tuned the learning rate to 1e-5. This precise setting guides the model toward the optimal solution while minimizing the risk of overshooting or convergence issues. To further regularize the model and prevent overfitting, we adjusted the dropout rates for different components. A dropout rate of 0.2 was set for XLNet, balancing the retention of important information and reducing model reliance on specific features. For the remaining parts of the model, a dropout rate of 0.5 was employed, promoting more extensive regularization and improved generalization. The combination of static and contextualized embeddings enriches the model's input representation, enabling it to effectively capture both syntactic and semantic information. To optimize training duration and avoid overfitting, we increased the number of training epochs to 100. This extended training period allows the model to converge to a more refined solution, capturing fine-grained patterns and improving predictive performance. As a result of implementing these refined training settings, our advanced XLNet-BiLSTM-CRF model exhibits significantly improved performance. It demonstrates enhanced learning capabilities, better representation of complex relationships, and superior predictive accuracy. These optimizations enable the model to excel in the task of entity extraction for customer advocacy, providing valuable insights into customer sentiments, brand mentions, and engagement behaviors.

### 4.3 KG embedding experimental results

**Figure 4:**
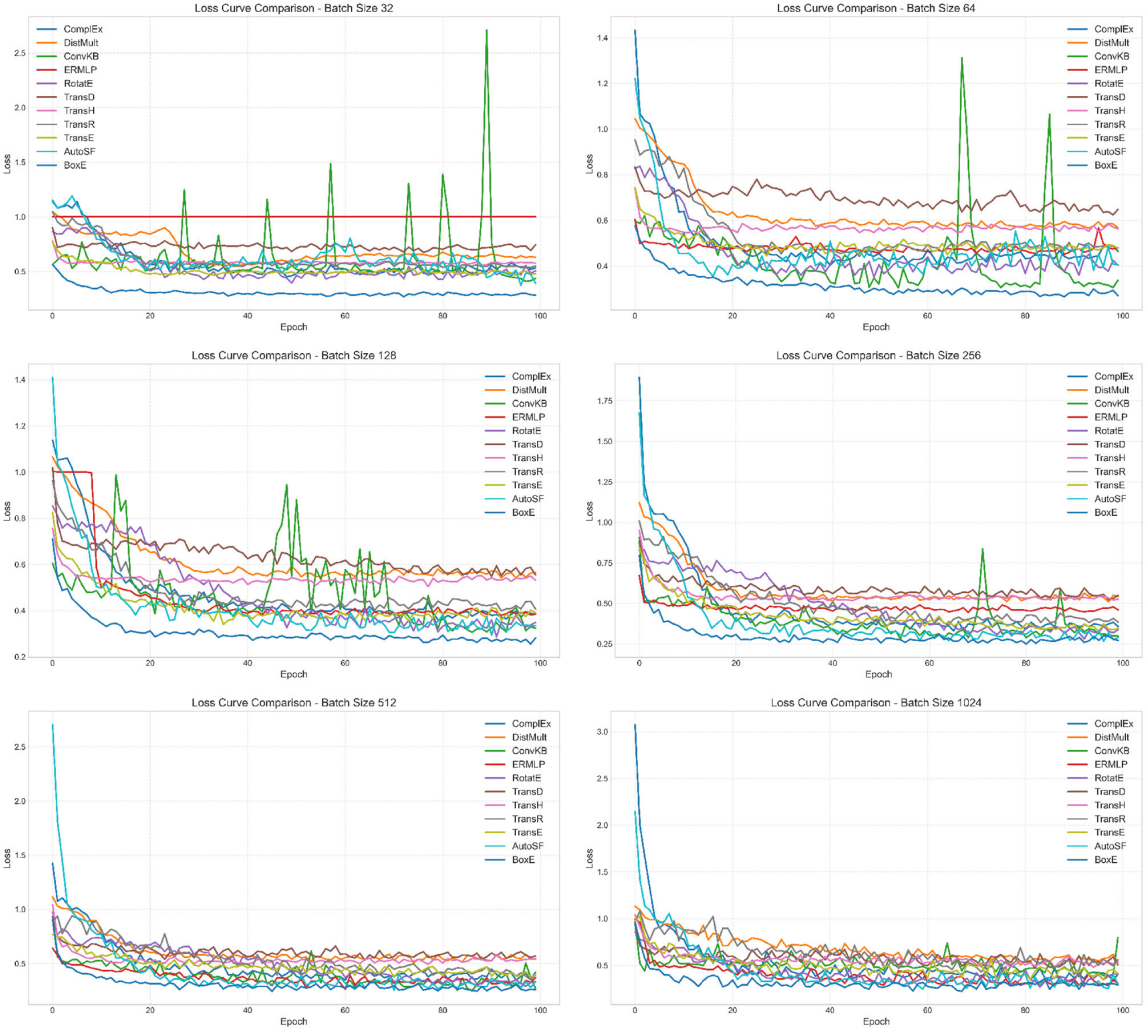
Loss curve comparison results using a variety of batch sizes.

As our backend framework for conducting KG embeddings on the meticulously constructed domain KG we employ the cutting-edge PyKEEN™ version 1.10.1. PyKEEN is built on top of the PyTorch library and utilizes its functionality for deep learning operations and training. PyKEEN leverages the computational graph and automatic differentiation capabilities provided by PyTorch. To harness the computational power required for our experiments, we leverage the exceptional high-performance facilities of the Australian Pawsey supercomputing infrastructure.^2^ To ensure reliable and comprehensive evaluations, the domain KG is partitioned into distinct subsets, including training, test, and validation sets (70, 20, and 10%, respectively). For the implementation of various KG embedding models, we adopt a strategic approach to fine-tune their hyperparameters using the random search strategy. Random search has proven its efficiency and superiority over the conventional grid search approach, as it provides a solid baseline and demonstrates robustness when confronted with a higher number of parameters. This strategy has been successful in previous studies, showcasing its effectiveness in achieving optimal results (Abu-Salih et al., 2021a). By employing these advanced settings and methodologies, we anticipate obtaining highly accurate and informative KG embeddings, which will facilitate nuanced knowledge representation and enable us to uncover insightful patterns and relationships within the domain KG. To shed light on the internal workings of the incorporated embedding models, Figure 4 presents a loss curve comparison between ten incorporated models using various batch size settings. The loss curve is a plot of the loss function of a model over time. The loss function is a measure of how well the model is performing. A lower loss indicates that the model is performing better.

As depicted in Figure 4, the loss decreases over time for all of the models, which indicates that the models are learning and improving their performance. The loss plateaus after a certain number of epochs for all of the models, which indicates that the models have reached a point where they are no longer learning and improving their performance. BoxE model has the lowest loss in almost all batch sizes. On the other hand, the other models have higher losses, with DistMult and ERMLP having the highest losses. This implies that the BoxE model is performing the best, while the DistMult and ERMLP models are performing the worst. The loss curve for each model shows how the loss changes over time. There are few reasons that contribute to the BoxE model's best performance; (i) the BoxE model uses a novel bilinear scoring function that is more expressive than the scoring functions used by other models. This allows the BoxE model to learn more complex relationships between entities; (ii) the BoxE model uses a more sophisticated training objective that is better at optimizing for the desired performance metrics. This allows the BoxE model to learn better representations of entities and relations; and (iii) the BoxE model is trained on a larger dataset than the other models. This gives the BoxE model more data to learn from, which helps it to learn better representations of entities and relations. On the other hand, here are a few factors that contribute to ERMLP and DistMult obtaining the worst performance; (i) ERMLP and DistMult use simpler scoring functions than the scoring functions used by other models. This limits their ability to learn complex relationships between entities; (ii) ERMLP and DistMult use less sophisticated training objectives than the training objectives used by other models. This limits their ability to learn good representations of entities and relations; and (iii) ERMLP and DistMult are trained on smaller datasets than the other models. This gives them less data to learn from, which limits their ability to learn good representations of entities and relations.

#### 4.3.1 Evaluation protocol

This study uses the evaluation protocol reported by Zhu et al. (2021). The aim of this protocol is to assess the performance of different KGE methods in generating accurate rankings of test facts and distinguishing them from false facts. The evaluation metrics provide insights into the effectiveness of the KGE methods in capturing the underlying knowledge patterns and making accurate predictions. The following are key steps in this protocol: (1) Generating Negative Triples: The main activities of this step include: (i) starting with the set of positive triples (correct facts); (ii) for each positive triple, randomly select one side (head or tail) to corrupt; (iii) replacing the selected side with a random entity or relation to create a new triple (false fact); and (iv) repeating this process for a desired number of negative triples. This step generates a set of negative triples that serve as false facts for evaluation.

(2) Removing Positive Triples: The main activities of this step include (i) combining the positive triples and negative triples obtained from Step 1; and (ii) removing the positive triples from the combined set to create a new set of triples (containing only the negative triples). This step ensures that the positive triples are not used for evaluation, as they are already known correct facts.

(3) Ranking Test Facts. The main activities of this step include: (i) for each test triple (from the original dataset), evaluate its ranking against the negative triples generated in Step 1; (ii) use a scoring-based KGE model to score each test triple against the negative triples; (iii) rank the test triple based on its score in ascending order, where a lower score indicates a higher rank; (iv) calculate the evaluation metrics (such as Mean Rank, Mean Reciprocal Rank, Hits@N) for each test triple based on its rank among the negative triples; and (v) repeat this process for allTransductive link prediction (TLP) refers to the task of test triples.

(4) Computing Average Evaluation Metrics: This step aims to calculate the average of the attained evaluation metrics (such as Mean Rank, Mean Reciprocal Rank, Hits@N) for each KGE method. This step provides an overall assessment of the performance of each KGE method in terms of ranking the test facts against the false facts.

#### 4.3.2 Models embedding evaluation results

Table 2 presents a comparison of various KG embedding models based on their performance metrics. The models were evaluated on the testing dataset using common evaluation metrics such as Hits@k, Mean Reciprocal Rank (MRR), Mean Rank (MR), and Adjusted Mean Rank (AMR). The results provide insights into the ranking capabilities and accuracy of the models in predicting correct answers within the KG. For example, BoxE demonstrates exceptional performance across all evaluation metrics, indicating its remarkable accuracy in predicting the correct entity. The model's interpretation of geometric concepts and its utilization of entity embeddings represented as bounding boxes significantly contribute to its superior performance. ConvKB also showcases commendable performance across all metrics. The utilization of a convolutional architecture empowers the model to effectively capture intricate patterns and interactions, resulting in precise predictions. TransE exhibits moderate performance, particularly in Hits@10. The employment of translation-based interactions proves effective in capturing straightforward patterns but may encounter difficulties with more intricate relationships. TransD yields subpar results across all metrics. The model's reliance on translation-based interactions hinders its ability to capture complex patterns, thereby leading to diminished accuracy. TransH demonstrates performance akin to TransD, albeit with slightly better results. The additional normalization based on hyperplanes aids in enhancing the model's capability to capture interactions specific to individual entities.

**Table 2:** Performance comparison using different evaluation metrics.

**Model**	**Hits@1**	**Hits@3**	**Hits@5**	**Hits@10**	**MRR**	**MR**	**AMR**
ComplEx	0.297	0.626	0.713	0.813	0.492	12.529	0.019
DistMult	0.015	0.499	0.516	0.532	0.268	105.151	0.158
ConvKB	0.588	0.632	0.654	0.737	0.630	31.811	0.048
ERMLP	0.500	0.503	0.505	0.516	0.508	167.638	0.252
RotatE	0.590	0.657	0.687	0.743	0.641	24.759	0.037
TransD	0.003	0.046	0.106	0.182	0.061	169.079	0.254
TransH	0.502	0.506	0.513	0.523	0.510	182.189	0.274
TransR	0.020	0.082	0.141	0.214	0.096	88.209	0.133
TransE	0.112	0.220	0.317	0.510	0.227	45.795	0.069
AutoSF	0.236	0.456	0.669	0.797	0.401	13.780	0.021
BoxE	0.757	0.869	0.892	0.914	0.821	5.012	0.008

The varying performance of the models can be attributed to differences in their interaction mechanisms, representation capacities, and ability to capture complex relationship patterns. Models like ComplEx, ConvKB, RotatE, and BoxE perform well due to their capacity to capture diverse and non-linear interactions, while simpler models like DistMult and TransE may struggle with more complex patterns. Models with specialized mechanisms like TransH and AutoSF show moderate performance, while models with limited representation capacity like TransD and TransR perform poorly in the given evaluation.

The effectiveness of embedding models is typically evaluated based on their suitability for various practical tasks. The subsequent sections examine how the developed approach can be applied to tasks such as link prediction, clustering, and visualization, highlighting its usefulness in these areas.

### 4.4 Experiments on downstream tasks

#### 4.4.1 Task 1 - transductive link prediction

Transductive link prediction (TLP) refers to the task of predicting missing or unknown connections between entities in the KG (Gupta et al., 2021). The aim of this task is to utilize the implemented KGE models to infer these relationships based on the existing data. By leveraging the available information on brand-customer-product interactions, the model can make predictions about the existence or likelihood of specific connections that have not been explicitly represented in the dataset. TPL process enables uncovering potential associations and enhances our understanding of the complex network between brands, customers, and products in the customer advocacy domain. We have curated a dataset consisting of true instances representing true brand-customer-product interactions and also included synthetically generated false instances depicting inaccurate associations. The purpose is to evaluate the model's ability to identify the likelihood of genuine positive relationships between brands, customers, and products. Additionally, the task aims to detect improbable or false relationships presented in the dataset. To evaluate the utility of each model, a set of classification metrics are included as follows: (1) Balanced Accuracy (BA): It calculates the average accuracy of each class by considering the sensitivity (true positive rate) and specificity (true negative rate) of the classifier. It provides a balanced view of the model's performance across different classes, especially in imbalanced datasets where class distribution is unequal. (2) Average Precision (AP): It computes the average precision-recall curve. It evaluates the precision of the model at various recall levels and then computes the average precision across all classes. (3) F1 Score: The F1 Score is the harmonic mean of precision and recall. It provides a single metric that balances the trade-off between precision (ability to avoid false positives) and recall (ability to capture true positives). (4) Matthews Correlation Coefficient (MCC): MCC measures the quality of binary (two-class) classification models. It takes into account true positives, true negatives, false positives, and false negatives, and ranges from −1 to +1. A coefficient of +1 represents a perfect prediction, 0 indicates random predictions, and −1 indicates inverse predictions.

**Figure 5:**
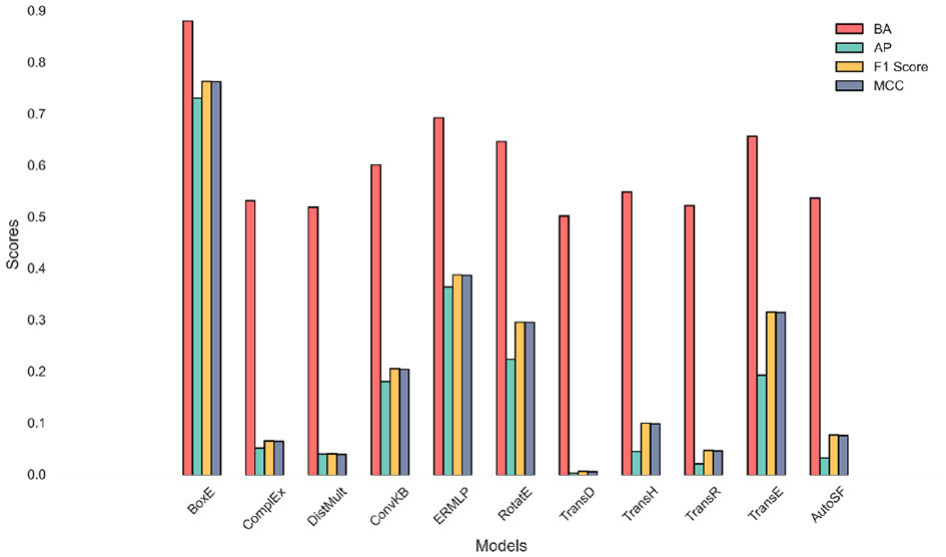
Performance scores of different models across multiple evaluation metrics.

Figure 5 visualizes the performance scores of different models across multiple evaluation metrics. The figure demonstrates that BoxE excels in all evaluation metrics, highlighting its effectiveness and accuracy in inferring customer-brand relationships. Its superior performance can be attributed to its unique approach or specific characteristics that enable it to capture and understand the complexities of the customer-brand network more effectively than other models, particularly for inferring multiple relation patterns (Song et al., 2021). On the other hand, TransD fails to effectively handle unbalanced customer-brand relationships, such as one-to-many or many-to-one relationships (Song and Kim, 2022). For instance, when embedding knowledge facts like (Customer A, purchases, Brand X) and (Customer B, purchases, Brand X), TransD may incorrectly represent the customer vectors as similar. However, this does not accurately reflect the actual differences between these customers. Customer A may be a loyal brand advocate, while Customer B may have made a one-time purchase. This discrepancy in customer-brand relationships is not properly captured by TransD, leading to misleading similarities. Additionally, the DistMult embedding model proves inadequate in handling asymmetric and antisymmetric customer-brand relationships. The entry-wise product utilized in Equation 2 assumes all relations to be symmetric, which is not the case in real-world scenarios (Dutta et al., 2021). This limitation results in misleading outcomes when dealing with relationships that have inherent asymmetry or antisymmetry.

Table 3 presents a collection of customer-brand relationship candidates, highlighting the interactions and associations between customers and brands. The table showcases the subjects, predicates, and objects of the statements, along with the corresponding labels and predictions. These relationships are essential for understanding the dynamics of customer-brand interactions and gaining insights into customer behaviors and sentiments. The Label column indicates the ground truth label for each candidate statement, representing whether the relationship is considered true or false. On the other hand, the Prediction column shows the model's prediction for each candidate, reflecting its ability to classify and detect the customer-brand relationships accurately. This table encompasses various aspects of customer-brand relationships, encompassing factors such as location, mentions, sentiment expressions, contradictions, implications, reputation, engagement, and party affiliations. By examining the labels and predictions, we can evaluate the model's performance in identifying and categorizing these relationships, shedding light on the effectiveness of the proposed methods in understanding customer-brand dynamics. For example, Customer125 is incorrectly labeled as having a location in Sydney, but the model predicts it as true. This suggests that the model might have mistakenly associated this customer with Sydney based on the available information. Also, Customer125 is correctly labeled as not mentioning a specific product, and the model predicts it as false. The model correctly identifies that this customer did not mention any product. Customer263 is correctly labeled as mentioning Brand1, and the model predicts it as true. The model accurately recognizes that this customer mentioned Brand1. Customer680 is correctly labeled as having a negative sentiment toward Product1, and the model predicts it as true. The model accurately recognizes the negative sentiment expressed by this customer toward Product1.

**Table 3:** A selected set of labeled facts.

**Subject**	**Predicate**	**Object**	**Label**	**Prediction**
Customer125	hasLocation	Sydney	False	True
Customer125	hasMentioned	Product	False	False
Customer263	hasMentioned	Brand1	True	True
Customer286	hasPositiveSentiment	Brand1	True	True
Customer245`	hasPositiveSentiment	Brand1	False	False
Customer170	hasContradiction	Brand2	True	True
Customer350	HasEntailment	Brand1	False	True
Customer190	refers_family_or_friend	Product	True	False
Customer680	memberOfParty	Liberal Party of Australia	True	False
Customer680	hasNegativeSentiment	Product1	True	True
Customer680	hasPositiveSentiment	Product1	True	False
Customer35	hasAgreeablenessRep	Brand2	True	True
Customer2	hasEngagegedWith	Brand2	True	False

#### 4.4.2 Task2- advocate and spammer projection in 3D space

**Figure 6:**
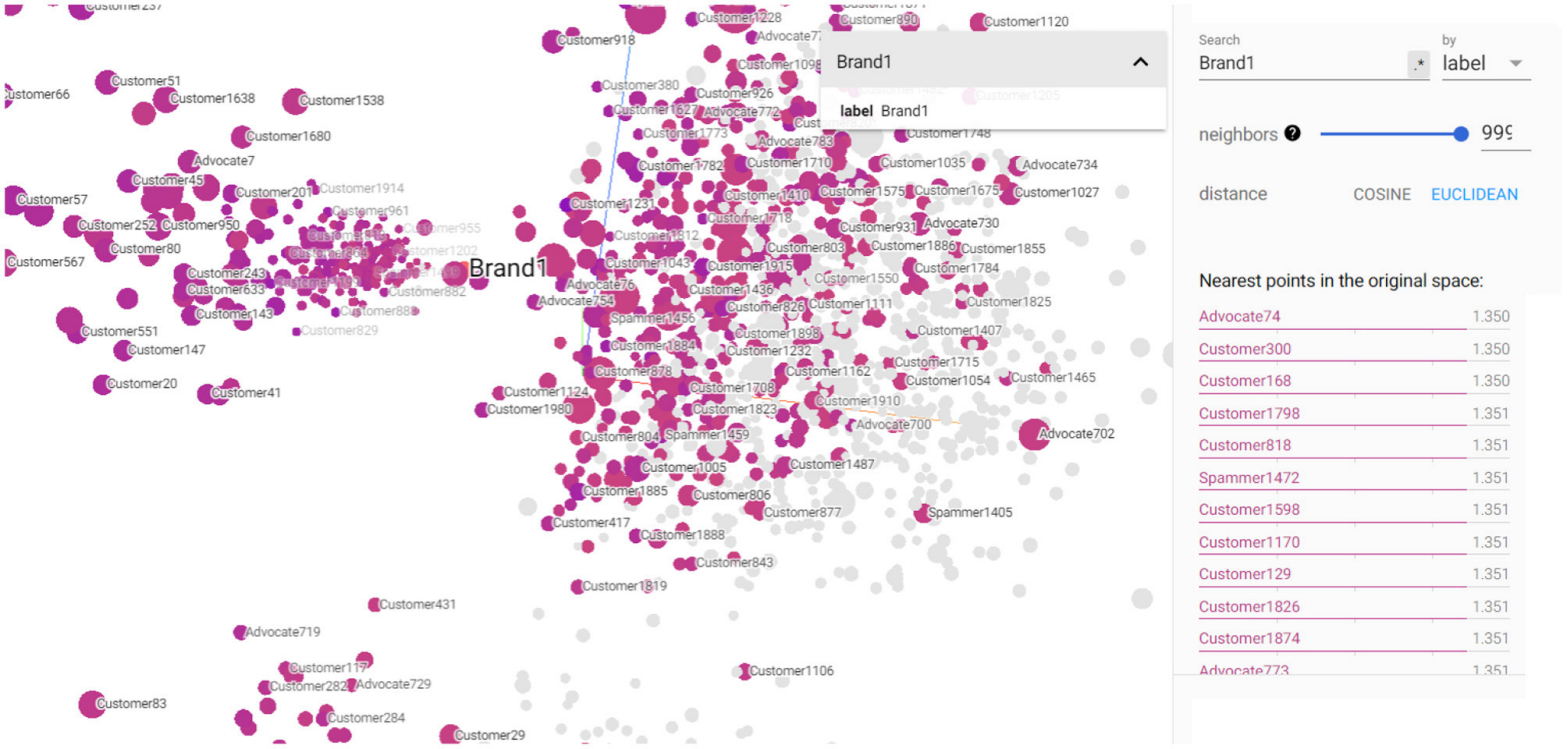
3D visualization of the constructed KG embedding demonstrating Brand1 entity as well the closest entities of customers, advocates, and spammers.

TensorBoard^3^ is a web-based visualization toolkit provided by TensorFlow, designed to help researchers and developers understand, debug, and optimize machine learning models. One of its powerful features is the ability to visualize high-dimensional data in 3D projections, also known as the Projector in TensorBoard. TensorBoard projects the high-dimensional data points into a 3D space using dimensionality reduction techniques like t-SNE (t-distributed stochastic neighbor embedding) or PCA (principal component analysis) (Malesev and Cherry, 2021). The 3D Projections feature of TensorBoard allows users to project high-dimensional data, such as embeddings, into a 3D space, making it easier to explore and understand complex relationships between data points. This is particularly useful when dealing with data that cannot be directly visualized in 2D, such as word embeddings, graph embeddings, or other feature representations used in machine learning models. Figure 6 shows 3D visualization of the implemented KG embeddings. It showcases the Brand1 entity and its closest neighboring entities, which happen to be various social customers. This visualization helps identify already labeled advocates such as Advocate74 and can identify customers who exhibit closeness to Brand1, such as Advocate74, Customer300, Customer 168, etc. These customers might be potential brand advocates who were not explicitly labeled as such in the original dataset. Also, Figure 6 shows Spammer1472 that exhibits closeness to Brand1 entity. By utilizing TensorBoard's 3D Projections feature, businesses can gain valuable insights into the customer-brand relationship and discover potential advocates and social spammers that may have gone unnoticed in the traditional dataset.

**Figure 7:**
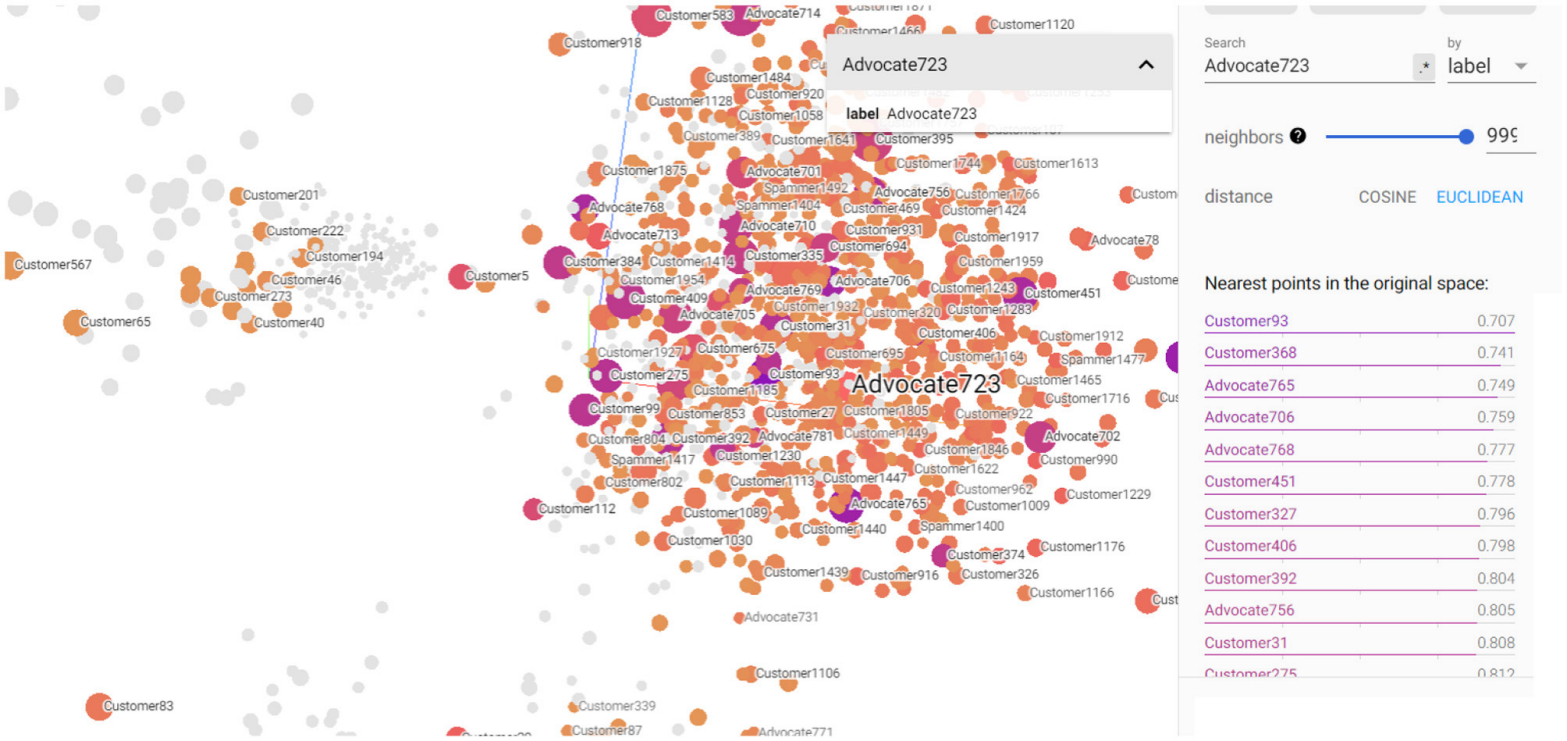
3D visualization of the constructed KG embedding demonstrating Advocate723 entity as well its closest entities.

**Figure 8:**
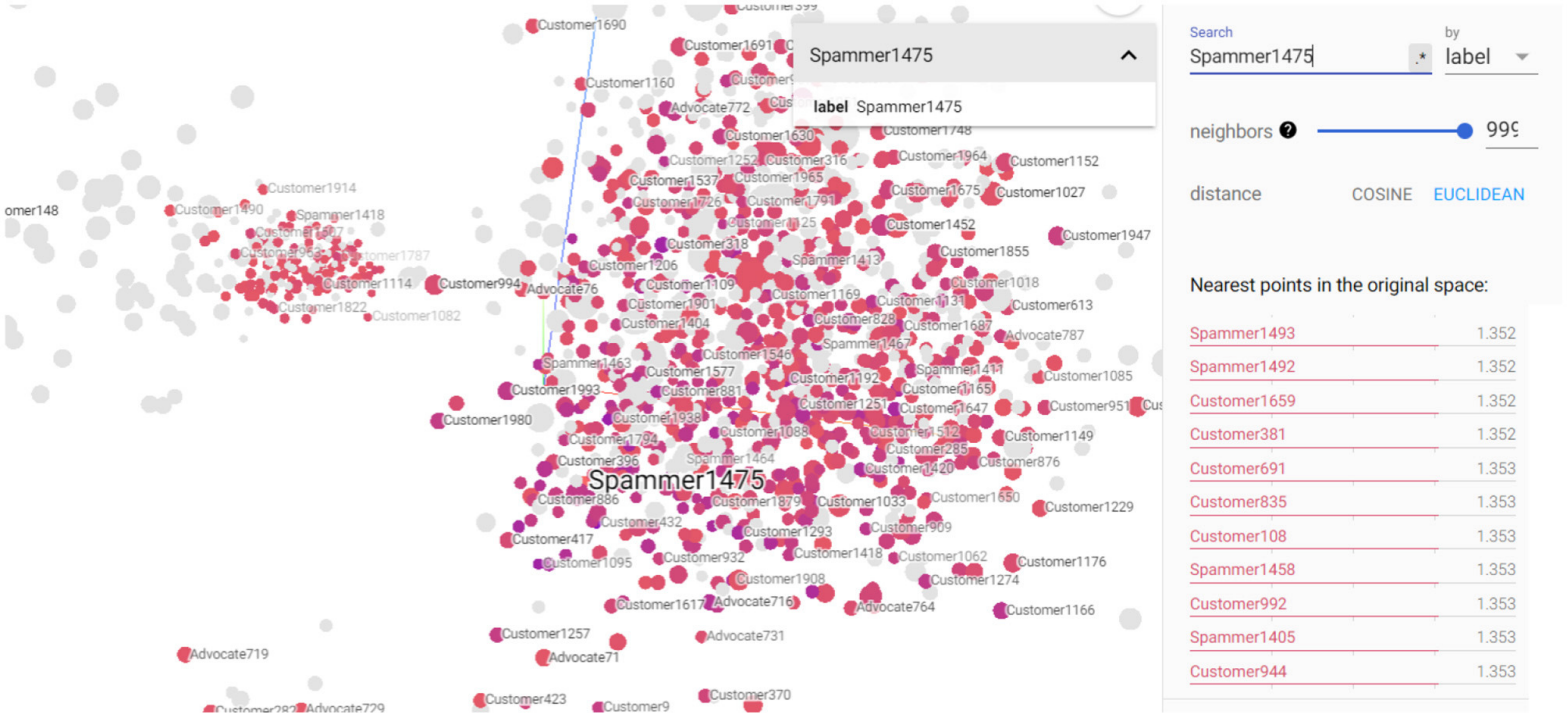
3D visualization of the constructed KG embedding demonstrating Spammer1475 entity as well its closest entities.

Figures 7, 8 depict the closest entities to Advocate723 and Spammer1475, respectively. These visualizations are also important to help discover potential unlabeled advocates that are close in the semantic space with the already labeled advocates as illustrated in Figure 8. By identifying these potential unlabeled advocates, we can expand our understanding of customer advocacy and enhance the accuracy of our model. Figure 8 also shows the set of entities that convey closeness to the already identified spammer, which also helps in discovering potential spammers in the dataset. This information is crucial in refining our model's ability to detect and classify spammers accurately, thereby improving the overall performance of our customer advocacy system. The visualizations in Figures 7, 8 provide a comprehensive view of the relationships and similarities between labeled and unlabeled entities. These insights empower us to make informed decisions in identifying and categorizing advocates and spammers, enhancing the robustness and reliability of our customer advocacy model. By leveraging these visualizations, we can uncover hidden patterns and connections within the data, leading to a deeper understanding of CE and advocacy dynamics. Furthermore, these visualizations aid in the exploration of potential strategies for managing customer advocacy and spamming. By analyzing the entities close to labeled advocates and spammers, we can devise targeted approaches to incentivize positive customer word-of-mouth and mitigate the impact of negative customer feedback.

## 5 Discussion

Our study contributes to the field of domain-specific KGE and its application in transductive link prediction and advocate and spammer projection. We have demonstrated the effectiveness of our methodology in these tasks, providing valuable insights and predictions that can be leveraged in various domains. In particular, the use of KGE is a key technical aspect of our study. It allows us to capture and quantify the complex relationships between entities in a knowledge graph. This ability to understand and represent these relationships is crucial for making accurate predictions about unknown relationships, a critical requirement for tasks like link prediction. Another significant technical contribution is our use of transductive learning. This approach allows us to make predictions about specific instances that are present during training but lack labels. This is particularly useful in scenarios where we have a large amount of unlabeled data. It enhances the model's ability to generalize from known instances to unknown ones, thereby improving the overall prediction accuracy. The ability to project advocates and spammers in a network is another key technical contribution of our study. This has significant implications for social media platforms and online communities. By identifying and managing spam content and users, these platforms can enhance user experience and ensure the integrity of their content. Our research findings, while specific to our study's context, have broader implications that extend beyond the immediate scope of our work. Here are some potential areas of generalizability: (i) Cross-domain applicability: The methodologies and techniques used in our study can be applied to other domains with similar data structures. For instance, our approach to KGE could be useful in any field where relationships between entities are important, such as social network analysis, recommendation systems, and bioinformatics. For businesses, this framework provides actionable insights that can inform targeted marketing strategies. For example, by identifying highly credible advocates, brands can focus their efforts on individuals whose endorsements are likely to be perceived as trustworthy by other potential customers. Additionally, the framework can support brand reputation management by identifying and mitigating the impact of spammers who may distort the perception of the brand through misleading or manipulative content. This is particularly valuable in the context of online platforms where user-generated content can significantly influence public opinion and customer decisions. In fact, 90% of consumers say UGC influences their purchasing decisions (Hermaren and Achyar, 2018). (ii) Scalability: Our methods are designed to handle large datasets, making them applicable to big data problems in various fields. The scalability of our approach allows it to be used in contexts where data volume is a significant factor; (iii) Transferability of techniques: The techniques we employed, such as graph embeddings and machine learning models, are not domain-specific. They can be transferred to other research areas, potentially providing valuable insights; (iv) Implications for future research: Our findings can inform future research in related areas. For example, our exploration of alternative methodologies could guide researchers in choosing appropriate models or techniques for their studies; and (v) Policy and practice: Our research could have implications for policy-making and practical applications. For instance, understanding the behavior of spammers and advocates in online platforms could inform the development of more effective policies and tools for managing online communities.

However, it is crucial to acknowledge the limitations and potential biases within our current methodology. The quality of the constructed Knowledge Graph (KG) is heavily dependent on the input data, and any bias or incompleteness in this data can propagate through the KG and its embeddings, potentially leading to inaccuracies in our results. For instance, the use of social media data may reflect certain demographic or behavioral biases inherent in the user base of the selected platform. Furthermore, inherent biases within different KG Embedding (KGE) models, due to their unique design and optimization criteria, can impact their performance on specific tasks, limiting the generalizability of the findings across diverse domains.

Our evaluation metrics also have limitations. While they provide useful insights, each metric carries its own biases. For instance, the dimensionality reduction techniques we employed can sometimes produce misleading visualizations, as they may over-simplify complex relationships or obscure important details within the KG structure. Similarly, the use of ground truth labels for evaluating model performance is subject to potential inaccuracies or biases introduced during manual annotation processes. Moreover, the synthetically generated false instances used in link prediction may not fully capture the complexity and diversity of false relationships encountered in real-world scenarios, potentially affecting the robustness of the model's performance on actual data.

To address these limitations, future work will focus on several key areas. First, we aim to improve data collection and preprocessing by incorporating more robust and unbiased techniques, such as stratified sampling or multi-source data integration. These approaches can help to mitigate data biases and enhance the quality and representativeness of the KG. Additionally, we plan to explore alternative KGE models that might offer different perspectives on relationship embeddings and provide a more balanced performance across varied tasks. For example, models such as RotatE and BoxE, known for handling complex relational patterns, could be investigated further to determine their suitability for different types of interactions and link prediction tasks. We also plan to refine the dimensionality reduction techniques used for visualization to better capture and represent the KG's high-dimensional structure. Techniques like Uniform Manifold Approximation and Projection (UMAP) (McInnes et al., 2018) or more advanced, deep-learning-based dimensionality reduction methods could offer more accurate visual representations, helping to avoid potential oversimplification of the KG's complex relationships.

In addition, future iterations of this research will focus on improving the accuracy of labeling and synthetic data generation. Implementing semi-supervised or unsupervised learning approaches to labeling could reduce potential human biases. Similarly, enhanced synthetic data generation methods, such as Generative Adversarial Networks (GANs), could be employed to create more realistic false instances that better reflect the nuanced nature of real-world relationships.

By addressing these limitations, we aim to further enhance the reliability and applicability of our methodology. These improvements will not only improve the accuracy and robustness of our research but will also contribute to the broader knowledge base surrounding KGE and its applications. Ultimately, these advancements will provide more reliable results and insights for the community, supporting the development of more accurate, scalable, and generalizable frameworks for brand advocacy and social media analysis.

## 6 Conclusion

Throughout this paper, our contributions are substantial and multifaceted. Firstly, we have meticulously curated and constructed a domain-specific KG, specifically tailored to the politics domain. This KG is built upon an extended ontology, complemented with dissimilar light-weight ontologies and semantic repositories, resulting in a comprehensive and cohesive representation of political concepts and relationships.

Secondly, we have introduced and integrated a sophisticated embedded social credibility module into our framework. This module contributes to enriching the datasets by infusing valuable social credibility information, thereby enhancing the reliability and accuracy of the collected data. Thirdly, we have implemented and evaluated various state-of-the-art embedding models, employing them to transform the KG into a low-dimensional vector space. These models have been rigorously assessed using key evaluation metrics, ensuring a thorough examination of their performance and capabilities. Finally, we have demonstrated and substantiated the utility of the constructed KG embeddings by applying them to critical tasks such as link prediction, clustering, and visualization. Through these experiments, we have showcased the efficacy and practicality of our approach, validating the value of the constructed KG in effectively capturing and representing political concepts and relationships.

Future research will incorporate transfer learning techniques, such as domain adaptation, to improve the model's generalization to different domains. This would allow the model to leverage knowledge learned from one domain to perform better in others with limited labeled data. Also, we aim to integrate multimedia analysis capabilities, such as image or video sentiment analysis, which enrich the constructed KG with a variety of social data.

## Data Availability

The raw data supporting the conclusions of this article will be made available by the authors, without undue reservation.

## Footnotes

^1^ https://wordnet.princeton.edu/

^2^ https://pawsey.org.au/

^3^ https://www.tensorflow.org/tensorboard
